# Hybridizing *Daphnia* communities from ten neighbouring lakes: spatio-temporal dynamics, local processes, gene flow and invasiveness

**DOI:** 10.1186/1471-2148-14-80

**Published:** 2014-04-12

**Authors:** Mingbo Yin, Sabine Gießler, Johanna Griebel, Justyna Wolinska

**Affiliations:** 1 School of Life Science, Institute of Biodiversity Science, Fudan University, Handan Road 220, Shanghai, China; 2 Department Biologie II, Evolutionsökologie, Ludwig-Maximilians-Universität, Großhaderner Str. 2, 82152 Planegg-Martinsried, Germany; 3 Department of Ecosystem Research, Leibniz-Institute of Freshwater Ecology and Inland Fisheries, Mueggelseedamm 301, 12587 Berlin, Germany

**Keywords:** Cyclical parthenogenesis, *Daphnia longispina* complex, Clonal richness, Microsatellite, Population structure

## Abstract

**Background:**

In natural communities of cyclical parthenogens, rapid response to environmental change is enabled by switching between two reproduction modes. While long periods of asexual reproduction allow some clones to outcompete others, and may result in “clonal erosion”, sexual reproduction restores genetic variation in such systems. Moreover, sexual reproduction may result in the formation of interspecific hybrids. These hybrids can then reach high abundances, through asexual clonal reproduction. In the present study, we explored genetic variation in water fleas of the genus *Daphnia*. The focus was on the short-term dynamics within several clonal assemblages from the hybridizing *Daphnia longispina* complex and the impact of gene flow at small spatial scales.

**Results:**

*Daphnia* individuals belonged either to the parental species *D. galeata* and *D. longispina*, or to different hybrid classes, as identified by 15 microsatellite markers. The distribution and genotypic structure of parental species, but not hybrids, corresponded well with the geographical positions of the lakes. Within parental species, the genetic distance among populations of *D. galeata* was lower than among populations of *D. longispina*. Moreover, *D. galeata* dominance was associated with higher phosphorous load. Finally, there was no evidence for clonal erosion.

**Conclusions:**

Our results suggest that the contemporary structure of hybridizing *Daphnia* communities from ten nearby lakes is influenced by colonization events from neighbouring habitats as well as by environmental factors. Unlike the parental species, however, there was little evidence for successful dispersal of hybrids, which seem to be produced locally. Finally, in contrast to temporary *Daphnia* populations, in which a decrease in clonal diversity was sometimes detectable over a single growing season, the high clonal diversity and lack of clonal erosion observed here might result from repeated hatching of sexually produced offspring. Overall, our study provides insights into spatio-temporal dynamics in a hybridizing *Daphnia* species complex in a recently established lake system, and relates genetic similarities of populations to a scenario of secondary invasion enhanced by environmental factors.

## Background

In natural communities, cyclically parthenogenetic reproduction is a complex and adaptable reproductive strategy with strong implications for ecological and evolutionary processes, such as genetic diversity, clonal erosion, interspecific hybridization and gene flow. In this mode of reproduction, one or more generations of parthenogenetically produced females alternate with a sexual generation formed by males producing sperm, and females producing haploid eggs. In cladoceran *Daphnia*, for example, females usually clone themselves by producing parthenogenetic daughters. Only when unfavourable conditions arise, such as food shortage, overcrowding, or change in temperature (e.g. [1]), do *Daphnia* individuals switch to the production of males and sexual haploid eggs that require fertilization, and then diapause. The general advantages of this reproductive mode are, on the one hand, enhanced genotypic diversity after sexual recombination and, on the other, high population growth rates during asexual periods (e.g. [2])*.*

The sexual phase of *Daphnia*’s reproductive cycle results not only in the production of new genotypes, but also in the formation of interspecific hybrids if closely related species co-occur (e.g. [3]). Via asexual propagation, these hybrids can then sometimes reach high abundances (e.g. [4,5]). Such hybridization is especially common among the members of the *Daphnia longispina* complex and widespread among the species *D. galeata* and *D. longispina* (taxonomy revised in [6]). Most previous work on spatial patterns in the *D. longispina* complex explored *Daphnia* communities across large geographical regions (e.g. [5,7]). For example, Keller et al. [5] found that although *D. galeata* and *D. longispina* are present on both sides of the Alps, *D. galeata* is more common in lakes south of the Alps, whereas *D. longispina* dominates in the north. In addition to this apparent geographical gradient, differences in environmental conditions also play a role [5]. However, little is known about spatial variation in the hybridizing *D. longispina* complex over small scales, where dispersal among neighbouring habitats might be of great importance. The main stage for *Daphnia*’s passive dispersal involves sexual diapause eggs being carried by water birds or transported by wind [8]. Since parental species produce more conspecific- than interspecific-crosses (therefore, more diapause eggs represent parental than hybrid genotypes), and since the “hatching success” of hybrid diapause eggs is inherently lower than of parental offspring [9,10], parental species are expected to have higher gene flow capacities than hybrids [10].

In cyclical parthenogens, long periods of strict asexual reproduction might result in declines of clonal diversity. This process has been described in life history models as “clonal erosion” [11,12]. In the field, such a decrease in clonal diversity over the growing season has been reported for populations of *Daphnia magna* (e.g. [13]) and of cyclically parthenogenetic rotifers (e.g. [14]). With respect to *Daphnia*, evidence for clonal erosion comes from populations inhabiting temporary ponds (e.g. [13]). In these habitats, sexual and asexual periods are strictly separated, with sexually produced offspring hatching from diapause eggs mainly in spring, giving rise to a large number of clonal lineages. Due to subsequent clonal competition, genetic diversity decreases gradually. In contrast, previous studies of *Daphnia* populations from permanent lakes did not detect a consistent decrease in clonal diversity over a growing season (e.g. [4,9]). In these and many other studies, however, the “clones” were defined by few allozyme loci and might actually have represented clonal groups instead of single lineages [15]. This methodological constraint could bias the results in such a way that a potential decrease in genotypic diversity might have been undetectable. Indeed, clonal erosion might also shape the genetic structure in overwintering *Daphnia* populations, as argued in a study applying higher resolution microsatellite markers [16]. However, this conclusion remains speculative, because it was derived from an analysis of genotypic structure of *Daphnia* populations only at the end of the growing season. In a more detailed summer-autumn field survey of the *D. longispina* complex in two large lakes, clonal diversity was traced over time by genotyping *Daphnia* at 10 polymorphic microsatellite loci [17]. This set of markers is able to detect true clonal lineages [18], and the clonal diversity was found to be roughly constant [17]. In that study [17], the exclusion of the spring period, during which thousands of new genotypes are expected to hatch from diapause eggs, may have prevented the detection of an initial drop in diversity (e.g. [13,19]). Consequently, to rigorously test for clonal erosion in permanent habitats, a survey must start early in the year when genetic diversity is expected to be highest, and changes must be followed over the whole growing season until late autumn, otherwise a decline might remain undetected.

In the present study, we explored spatial patterns and temporal dynamics in the genotypic structure of *Daphnia* communities from several lakes inhabited by taxa of the *D. longispina* complex. This was undertaken over a small spatial scale, with study sites separated by 30 km at most. We applied high-resolution genotyping (15 microsatellite loci). Specifically, we tested if the relative frequencies of parental species and their hybrids correspond with the geographical proximity and/or environmental descriptors of the lakes. Then, we looked for changes in taxon composition between spring and autumn seasons. In addition, we estimated gene flow and compared genotypic similarities among populations belonging to either parental species or hybrids, in order to find evidence for invasiveness and/or local production of hybrid taxa. Finally, some lakes were tracked throughout one growing season to determine if clonal diversity decreases over time (i.e., clonal erosion).

## Results

### Taxon assignment

**Figure 1:**
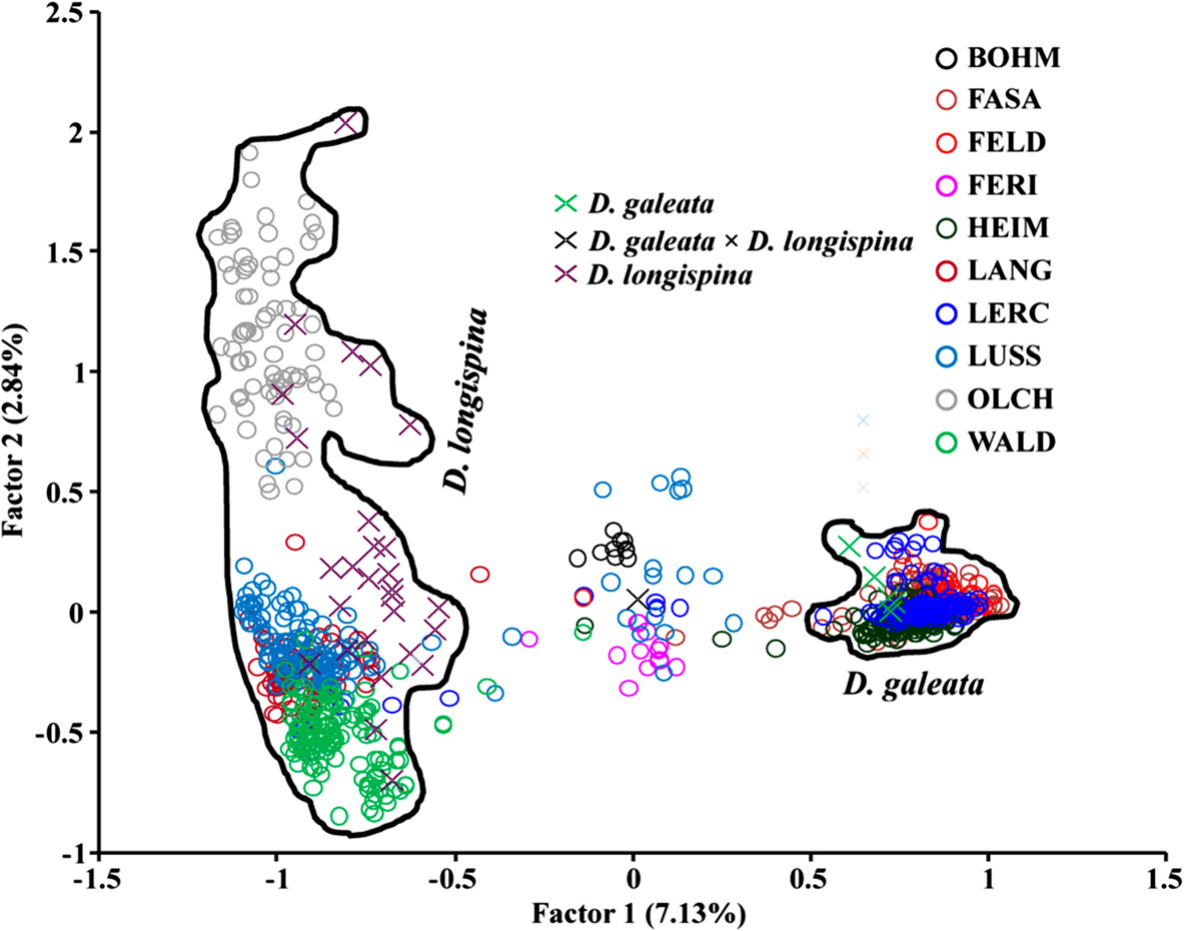
**Factorial correspondence analysis displaying genetic similarities between individuals from the *D. longispina *complex sampled in 2011 from ten lakes in and around Munich, based on allelic variation at up to 15 microsatellite loci (data from monthly samples are pooled).** For lake abbreviations see Additional file 1: Table S1. The 35 reference clones representing two parental species and their interspecific hybrids (indicated by crosses) are also shown (for a list of all reference clones see Table S1, Supporting information in Yin et al. 2010) [18]. The two outlines enclose the parental taxa as assigned by NewHybrids.

The 1934 *Daphnia* sampled from ten small lakes (flooded gravel pits) in and around Munich belonged to *D. galeata*, *D. longispina* or *D. galeata* × *D. longispina* hybrids. This was based on their position in the factorial correspondence analysis (FCA) in relation to the clusters formed by 49 well-defined reference genotypes representing three parental species or their respective hybrids (data not shown). To increase the resolution among genotypes, a separate FCA was run using reference genotypes from *D. galeata*, *D. longispina* and *D. galeata* × *D. longispina* hybrids (35 genotypes). The FCA revealed that *D. galeata* originating from four lakes (FASA, FELD, HEIM and LERC; for abbreviation see Additional file 1: Table S1) clustered together. Meanwhile, *D. longispina* from OLCH formed a separate cluster in relation to *D. longispina* originating from four other lakes (LANG, LERC, LUSS and WALD; Figure 1). Taxon identity was further confirmed by NewHybrids [20], which assigned *Daphnia* genotypes to four of six possible classes: *D. galeata* (47.9%), *D. longispina* (41.3%), F1 (9.4%) and F2 hybrids (0.8%); no backcrosses were detected. Eleven individuals (0.6%) could not be assigned, within a given 95% posterior probability, to any of the classes (Additional file 1: Table S1).

### Spatial patterns in taxon composition

**Figure 2:**
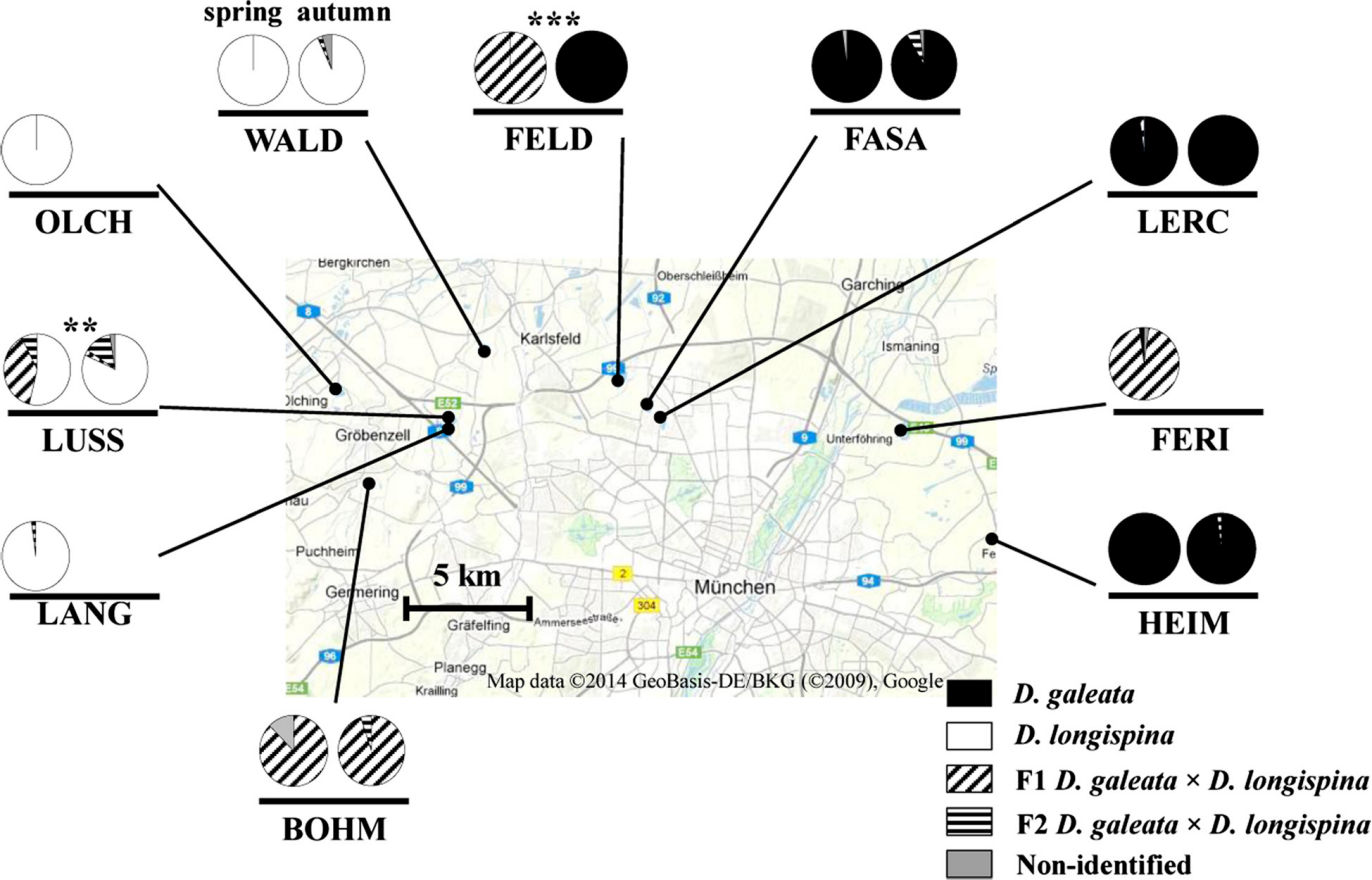
**Spatio-temporal variation among communities of the *D. longispina *complex, sampled in 2011 from ten lakes in and around Munich (for detailed lake characteristics see Additional file **2**: Table S2).** Seasonal changes in taxon composition are shown between spring (April or May; left pie charts) and autumn samples (October or November; or, in case of FELD, September; right pie charts). *Daphnia* individuals are assigned to taxa by NewHybrids, based on variation at up to 15 microsatellite loci. Significant differences between seasons are denoted by ** (*P <* 0.01), and *** (*P* < 0.001; after sequential Bonferroni correction) above the respective pie charts.

Across the sampling period (i.e. April – November 2011), only one parental species was present within a given lake, with the exception of FASA May sample (98% *D. galeata* and 2% *D. longispina*, N = 48) and LERC September sample (80% *D. galeata* and 20% *D. longispina*; N = 41, Additional file 1: Table S1). These were, however, the single sampling dates when *D. longispina* was detected in these otherwise *D. galeata*-dominated communities. Notably, there was a significant relationship between geographical distance and differences in the spring taxon composition, when calculated for presence/absence data (r = 0.23, *P* = 0.044), but not for frequency data (r = 0.18, *P* = 0.084). Specifically, the lakes north and east of Munich were dominated by *D. galeata* or hybrids (FASA, FELD, FERI, LERC and HEIM), whereas the lakes north-west and west of Munich were dominated by *D. longispina* or hybrids (BOHM, LANG, LUSS, OLCH and WALD; Figure 2).

### Environmental preferences of parental taxa

**Figure 3:**
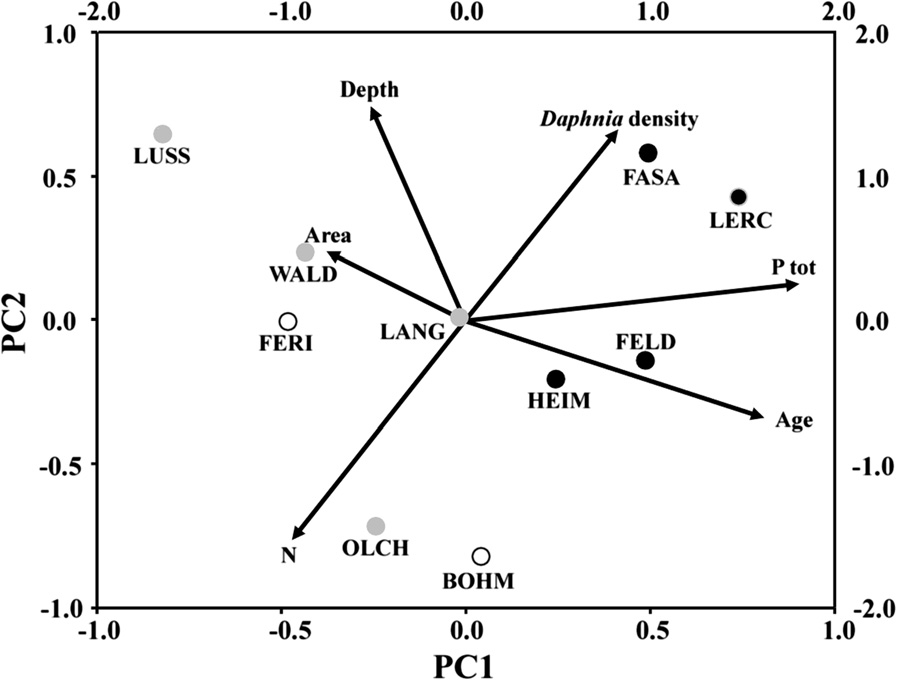
**Association of *Daphnia *species distribution to mean *Daphnia *density and environmental descriptors of ten sampled lakes.** The PCA plot displays (1) the projection of the original variables (see Additional file 2: Table S2) on to the first two components (coordinate scales: bottom and left) and (2) respective factorial loadings of lakes (coordinate scales: top and right). Arrows point to increasing importance for the respective variables. Each dot represents a lake. For lake abbreviations see Additional file 1: Table S1. Black dots: lakes inhabited by *D. galeata*; grey dots: lakes inhabited by *D. longispina*; white dots: neither parental species was detected (only hybrids were present). FASA and LERC (black dots with grey ring) are the only lakes where both species were detected. However, *D. longispina* was present at low frequencies: 0.3% (1 individual) and 2.5% (8 individuals), respectively – all samples pooled.

Two main principal components were extracted from six variables (physical and environmental characteristics; Additional file 2: Table S2) by eigenvalue > 1. The first component PC1 (explaining 38.1% of the variance) reflected mainly the trophic status and age of the lake; the higher the scores, the more eutrophic (i.e., high P_tot_) and older the lakes were. The second principal component PC2 (explaining 29.6% of the variance) was associated mainly with high lake depth and low nitrogen concentration (Figure 3). Overall, lakes inhabited by different *Daphnia* species were characterized by opposite component loadings on the first principal components analysis (PCA) axis. Thus, lakes inhabited by *D. galeata* were more eutrophic and older (df = 1, F = 19.99, *P* = 0.002), while most lakes inhabited by *D. longispina* were of lower trophic level and younger (Figure 3). Moreover, when FASA and LERC were scored as lakes without *D. longispina* (*D. longispina* was present at low frequencies; 0.3% (1 individual) and 2.5% (8 individuals), respectively – all samples pooled), the difference in mean component loadings on the PC1 axis was significant for this species also (F = 6.01, *P* = 0.04; otherwise: F = 0.97, *P* = 0.35). In contrast, neither lake depth nor nitrogen concentration (loadings on PC2) had any influence on species abundance (*D. galeata*: F = 0.71, *P* = 0.42; *D. longispina*: F = 0.54, *P* = 0.35; see Figure 3).

### Temporal changes in taxon composition

Over time, *Daphnia* taxon composition changed significantly between spring and autumn in two of the seven lakes tested. In FELD, the community changed from complete dominance by F1 hybrids to complete dominance by *D. galeata*; in LUSS, *D. longispina* increased its abundance in comparison to the F1 hybrids (Figure 2).

### Genetic distances among spatially and temporally isolated populations of parental species and hybrids

**Figure 4:**
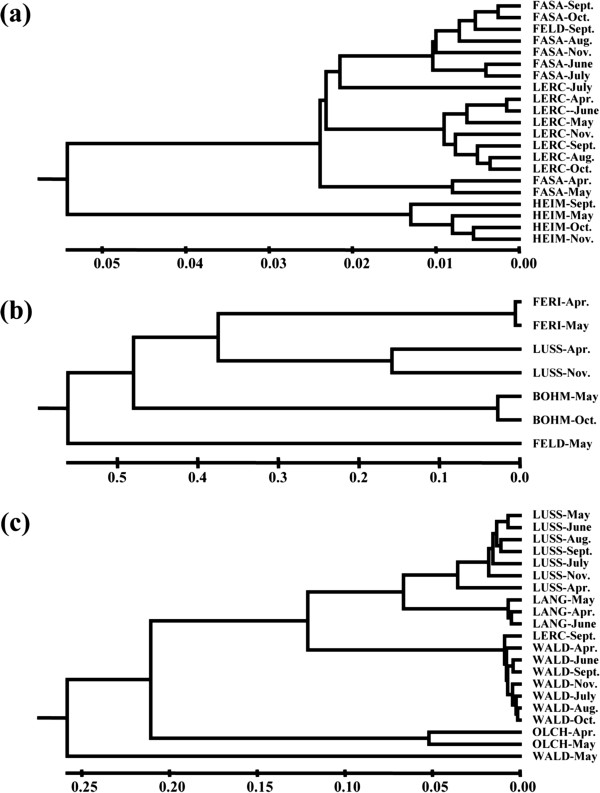
**UPGMA clustering of Nei’s genetic distances among populations of: (a) *D. galeata*, (b) hybrids (including F1 and F2 individuals) and (c) *D. longispina*, as sampled in 2011 from ten lakes in and around Munich.** Monthly samples are shown separately. The genetic distance was calculated based on variation at up to 15 microsatellite loci.

Similarities among populations from different lakes and sampling dates are illustrated by Unweighted Pair-Group Method with Arithmetic Mean (UPGMA) dendrograms based on Nei’s genetic distance [21]. In general, populations from the same lake (although sampled in different months) clustered together (Figure 4). There were four exceptions to this pattern: *D. galeata* from LERC July sample, *D. galeata* from FASA April and May samples (Figure 4a), and *D. longispina* from WALD May sample (Figure 4c). These particular samples were distinct from others from the respective lakes, because the genotypes which were dominant in these samples were not present in other periods (Additional file 3: Figure S1). For *D. galeata* and *D. longispina*, the genetic similarities among populations corresponded well with the geographical distribution of populations. Specifically, *D. galeata* from FASA, FELD and LERC (i.e., the lakes within a distance of 500 m, see Figure 2) clustered together but were otherwise isolated from HEIM, a lake which is 15 km away (Figure 4a). *D. longispina* from LUSS and LANG (lakes only ~100 m apart) clustered, but were isolated from *D. longispina* from LERC, WALD or OLCH, lakes separated by a distance of > 6 km (Figure 4c). In contrast, the clustering of hybrid populations did not correspond to their geographical position: hybrids from FERI and LUSS were the closest on the dendrogram, but the most isolated geographically (Figure 4b). Overall, the genetic distance between the most isolated populations within a given taxon was highest for hybrids (~0.55), half as great for *D. longispina* (~0.25), and one-tenth as great for *D. galeata* (~0.055, Figure 4).

### Gene flow and local production of hybrids

The mean number of migrants among lakes (all pairwise comparisons per taxon) differed between the parental species and hybrids (df = 2, F = 4.73, *P* = 0.023). Specifically, there were significantly more migrants among *D. galeata* populations (Nm = 4.26, SD = 4.63, N = 6) than among *D. longispina* (Nm = 0.84, SD = 0.28, N = 9) or hybrids (Nm = 0.37, SD = 0.10, N = 6), meaning that gene flow was largest for *D. galeata* (Post-Hoc test: *P* < 0.05)*.* Then, evidence for local production of hybrids was found. Specifically, the hybrids from LUSS (i.e. a lake where hybrids coexisted with parental species) were more likely assigned to the respective local parental *D. longispina* population than to populations of *D. longispina* from other lakes (df = 17, t = -14.2, *P* < 0.001).

### Test for clonality

Among 1856 individuals with complete multilocus genotypes (MLG) profiles (or data missing solely at the SwiD2 locus) 867 unique MLGs were detected. The hypothesis that individuals sharing identical MLGs were of sexual origin was rejected (*P* < 0.001 in all cases), indicating that individuals with identical MLGs can be treated as the same clone.

### Temporal changes in clonal composition

**Figure 5:**
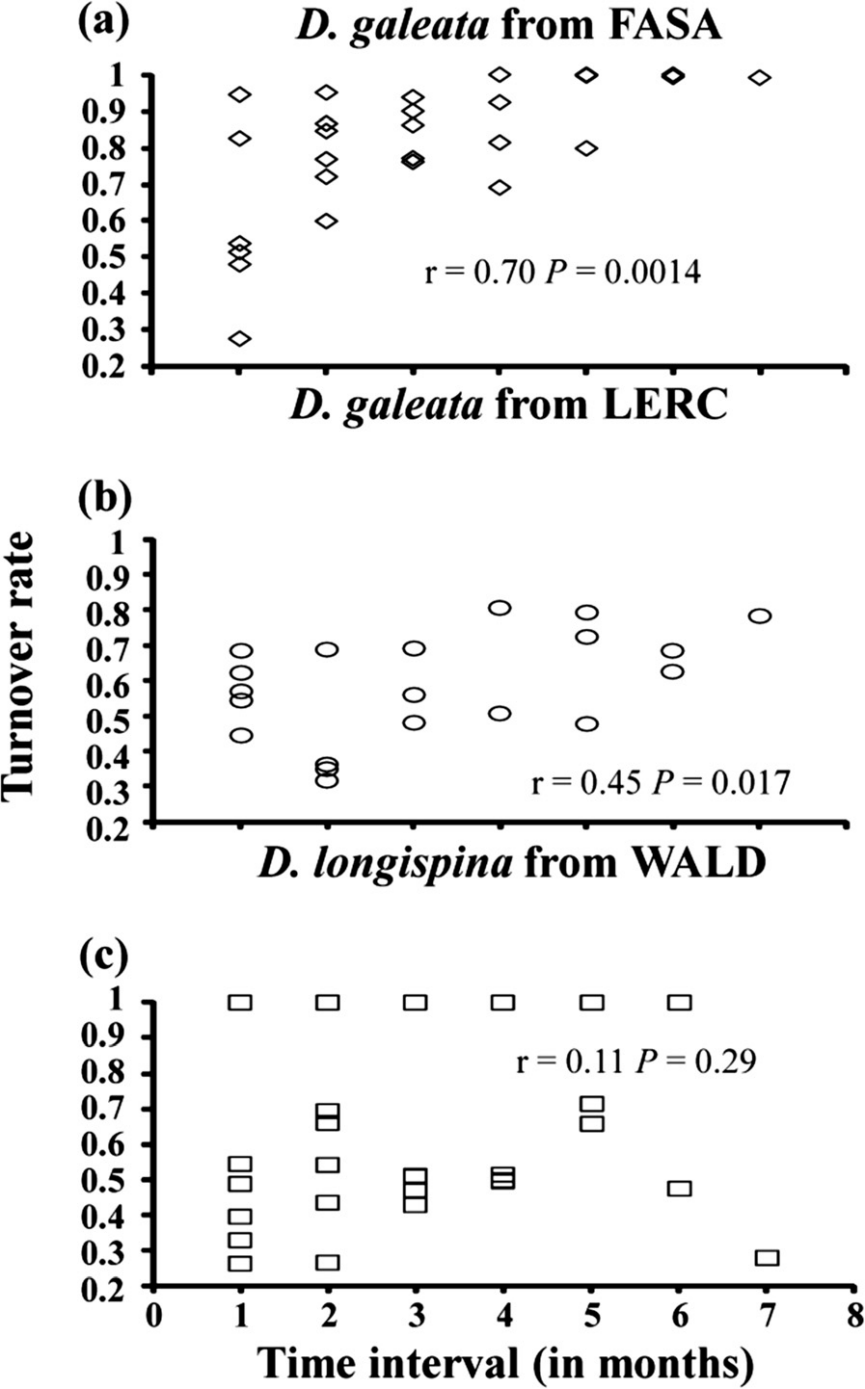
**Relationship between sampling time intervals and genotype turnover rates (1 - Morisita-Horn index) for: (a) *D. galeata *from FASA, (b) *D. galeata *from LERC and (c) *D. longispina *from WALD (only in these three lakes were *Daphnia *communities present throughout the entire sampling season, i.e. from April to November 2011, and the sample size per analysed population** ≥ **30).** The turnover rate varies from 0 (complete similarity) to 1 (no shared MLGs). The Pearson correlation coefficient is shown for each analysis. Genotypes were assigned based on variation at all 15 microsatellite loci (or 14 loci, if *Daphnia* could not be amplified at locus SwiD2).

**Figure 6:**
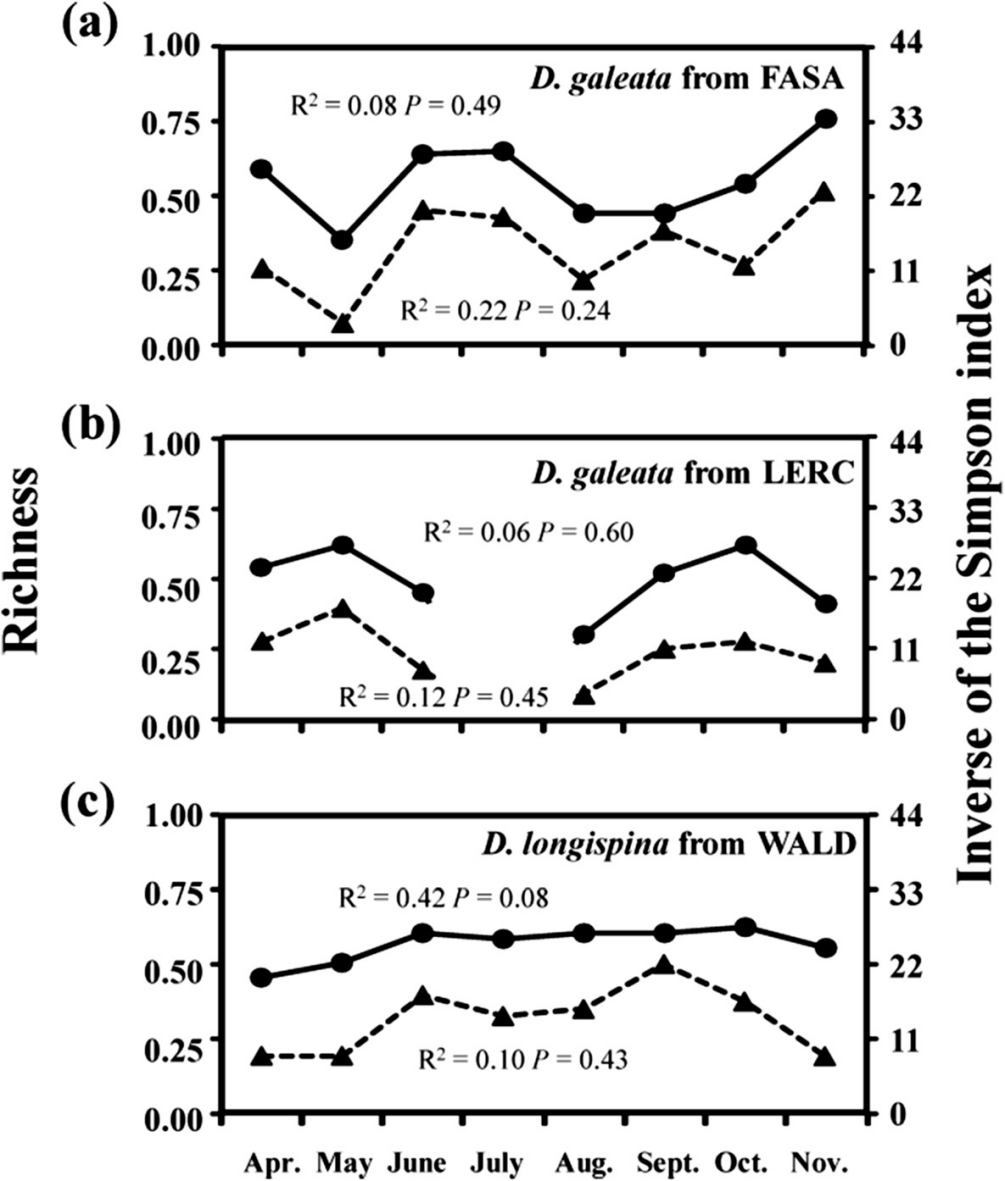
**Temporal changes in clonal diversity, calculated as clonal richness R (solid line) or as the inverse Simpson index (dashed line) for (a) *D. galeata *from FASA, (b) *D. galeata *from LERC and (c) *D. longispina *from WALD (only in these three lakes were *Daphnia *communities present throughout the entire sampling season, i.e. from April to November 2011, and the sample size per analysed population** ≥ **30).** The upper/lower equation shows the results of linear regression analyses between time and clonal richness/inverse of the Simpson index, respectively. Missing data indicate a month when too few animals were available to calculate genetic indices of clonal diversity. The genetic indices were calculated based on variation at all 15 microsatellite loci (or 14 loci, if *Daphnia* could not be amplified at locus SwiD2).

The most striking change was observed in FELD, where in May the community was dominated by only one hybrid clone. After the community crash, hybrids disappeared and multiple *D. galeata* clones were detected in September (Additional file 1: Table S1). Regarding the three populations for which it was possible to study changes in clonal composition in more detail (i.e., *D. galeata* from FASA and LERC, and *D. longispina* from WALD; samples available from April to November), a few common clones were present over a longer period of time, however the frequencies of these clones fluctuated greatly (Additional file 3: Figure S1). Furthermore, differences in clonal composition increased significantly with the time interval between sampling dates (i.e., indicating replacement of clones) for *D. galeata* from FASA (Figure 5a) and *D. galeata* from LERC (Figure 5b), but not for *D. longispina* from WALD (Figure 5c). There was no indication of clonal erosion in any of these three populations; genetic diversity (as measured by two indices: clonal richness R and the inverse of the Simpson index) did not decrease over time (Figure 6).

## Discussion

In the present study, we surveyed spatio-temporal dynamics in hybridizing communities of *Daphnia* originating from ten neighbouring lakes. This is a first insight into genetic diversity, clonal erosion, hybridization and gene flow on such a small spatial scale which is tightly linked to the adaptable reproductive strategy in species complexes formed by cyclical parthenogens.

### Spatial pattern and importance of environmental factors on taxon composition

A geographical separation was observed between communities dominated by *D. galeata* or *D. longispina* despite presumably ongoing dispersal between the neighbouring lakes. In a first step, lakes have been possibly colonized by extant neighbouring populations in the past. For example, Lußsee (LUSS) could have been colonized by *D. longispina* from Langwieder See (LANG), a lake created six decades earlier, and only 100 m away (see Figure 2). In *Daphnia*, and other zooplankton species, the advantage of first arrivals is relatively strong, due to their rapid population growth rate (e.g. [22,23]). Indeed, from studies of the colonization of artificial ponds, it has been reported that *Daphnia* populations were founded by only a few genotypes [24,25]. Moreover, after initial colonization, no new alleles were observed in these populations, indicating limited establishment success for genotypes arriving later [25]. However, it has also been observed that changes in environmental conditions might result in species shifts, especially in the *D. longispina* communities (e.g. [26]). Thus, independent from geographical proximity, differences in lake environmental conditions may explain part of the observed spatial variation in taxon composition. Indeed, PCA and corresponding statistical tests showed that *D. galeata* occurred in more eutrophic (and older) habitats, and *D. longispina* in less eutrophic (and younger) ones. This pattern is consistent with a postulate of Flößner and Kraus [27] that *D. galeata* is promoted by eutrophication. Moreover, by comparing the physical parameters and water quality of several lakes, Keller et al. [5] concluded that *D. longispina* prefers habitats with low phosphorus load. Species-specific contrasting preferences were confirmed by the detection of a gradual replacement of *D. longispina* with *D. galeata* during periods of eutrophication [26], and by follow-up experimental tests showing differential fitness of these two parental species under various phosphorus loads [28].

Surprisingly, although *D. galeata* occurred exclusively in the oldest lakes, the genetic distance among populations of *D. galeata* was ~1/5^th^ of that among populations of *D. longispina*, implying lower divergence time for *D. galeata*. This could possibly be explained by the fact that *D. galeata* spread later and, being more invasive and a better colonizer as suggested by the highest number of migrants, replaced former residents. Assuming that newly established artificial lake habitats are oligotrophic in the beginning and therefore first colonized by *D. longispina*, eutrophication might have allowed *D. galeata* to invade (see [26]). Overall, in our system the geographical separation between *D. galeata* and *D. longispina* dominated communities is consistent with the environmental conditions of the occupied habitats. Thus, geographical and environmental forces are not exclusive, and both most likely play a role in shaping *Daphnia* hybridizing communities.

### Temporal pattern and lack of clonal erosion

The relative proportion of *Daphnia* taxa changed significantly between spring and autumn in two of the seven tested lakes. In addition, there was significant replacement of clones over time in two out of the three lakes for which a complete set of time series data was available. The observed changes may be due to the impact of environmental variation on the relative fitness of parental species and hybrids, as well as the fitness of specific clones within species, as has been shown in previous experimental surveys (e.g. [29-31]). However, although extinction of clones due to selection and random events should lead to an overall decrease in clonal diversity towards the end of the growing season [11], no signs of clonal erosion were detected in our study. Consistent with previous reports for permanent lakes (e.g. [4,9]), in our system the indices for clonal diversity remained roughly constant over the growing season, both for *D. galeata* and *D. longispina* populations (hybrids were not tested because of their small sample size). The presence of clonal erosion in *Daphnia* populations may thus depend on the habitat type [11,32]. Specifically, in contrast to temporary pond populations with clear separation of sexual and asexual phases in the *Daphnia* reproductive cycle (e.g. [33]), and for which the importance of clonal erosion has been documented (e.g. [13,34]), the two modes of reproduction often overlap in the larger permanent lakes (e.g. [9,10,35]). This can contribute to the maintenance of high clonal diversity throughout the year. Moreover, given that fitness of genotypes in the *D. longispina* complex varies strongly with environment (e.g. [30,31]), seasonal environmental changes may favour the maintenance of a high number of genotypes through fluctuating selection pressures.

### Local production of hybrids

In contrast to parental species, neither the frequency of hybrids in the community, nor the clonal composition of hybrid populations was associated with the geographical neighbourhood of the lakes. Also, the UPGMA analysis showed that the highest genetic distance was detected among populations belonging to hybrids. Gene flow among hybrids was negligible and hybrids were more related to parents from the same lake than to parents from other lakes. These results complement evidence from previous studies showing that *D. galeata* × *D. longispina* hybrids have impaired hatching success from diapause eggs [9,10], possibly restricting their gene flow. This all suggests that these hybrids are rather locally produced, by multiple hybridization events [36].

## Conclusions

In summary, by applying high-resolution microsatellite markers, we addressed spatial and temporal patterns in the hybridizing *D. longispina* complex on a small scale, in ten recently established lakes. We found that the distribution and genotypic structure of parental *Daphnia* species was consistent with the geographical position and the differences in environmental attributes of the lakes; in particular, the presence of *D. galeata* was related to higher phosphorous load. Furthermore, the genetic distance among populations of *D. galeata* was lower than among populations of *D. longispina*, implying lower divergence time for *D. galeata*. The explanation for this may be that *D. galeata* spread later, being more invasive and a better colonizer, and then replaced former residents in lakes with high trophy level. Finally, no clonal erosion was detected across the studied lakes, suggesting that this phenomenon may depend on the habitat type and the extent of hatching of sexual offspring. Overall, the *Daphnia* system is a useful model to gain a better understanding of dynamics in hybridizing communities formed by cyclical parthenogens.

## Methods

### Ethics statement

Local administration has been contacted at the beginning of the study. Collection of zooplankton (*Daphnia*) did not require specific permissions as these samples were obtained from unprotected lakes that are open for public activities. Our study did not involve the use or collection of endangered or protected species.

### Study sites and field sampling

*Daphnia* individuals were sampled from ten flooded gravel pits in and around Munich (Germany; Additional file 1: Table S1). The lakes were created between 1930 and 2000, most are small and shallow, and the maximum distance between them is about 30 km. Geographical coordinates, lake age, lake size, maximum depth, total phosphorus concentration (P_tot_), nitrogen concentration (N) and *Daphnia* densities (averaged across monthly densities from April to November 2011) are given in the supplementary material (Additional file 2: Table S2). The phosphorus and nitrogen data were provided by Andreas Scholz from the Wasserwirtschaftsamt München (http://www.wwa-m.bayern.de/). These measurements were taken in spring, from the surface to the bottom of the lake at one-meter intervals (average P_tot_ and N values were then calculated). Zooplankton samples were collected monthly from April to November 2011 (eight samples per lake; sampling interval was about 30 days). A 95-μm plankton net was hauled through the whole water column at two different sites per lake within the deep basin (depth was measured using a portable depth sounder); the two samples were then pooled and preserved in 95% ethanol. A random subsample was used for density counts (only adult females were taken into consideration). Then, ca. 50 adult females from the *D. longispina* complex were chosen randomly per sample for genotyping. Since *Daphnia* densities were sometimes very low, a complete time series of eight samples could be genotyped for only three lakes (FASA, LERC and WALD; for lake abbreviations see Additional file 1: Table S1). Two to seven samples were genotyped for the seven remaining lakes, resulting in 1934 *Daphnia* individuals being genotyped in total.

### Microsatellite genotyping

The DNA of each individual *Daphnia* was extracted and genotyped at 15 microsatellite markers [37], in two multiplex polymerase chain reactions, following a protocol described elsewhere [18]. PCR products were analysed on an ABI PRISM 3700 capillary sequencer using a LIZ 500 labelled size standard. Genotypes were scored using GeneMapper version 3.7 (Applied Biosystems). Alleles at each locus were defined by their fragment length (in base pairs). Across different runs of genotyping, the consistency of alleles was checked, with locus-specific patterns of one reference genotype used in each run. This enabled us to adjust alleles with small differences in fragment length among different runs. In addition, there was no evidence of scoring errors due to stuttering, large allele dropout, or the presence of null alleles [38], as indicated by respective tests using MICRO-CHECKER 2.2.3 (10^4^ permutations, [39]).

### Taxon assignment

To display the genetic relatedness of multilocus genotypes (MLGs), factorial correspondence analysis (FCA) was applied in GENETIX 4.05 [40], on all unique MLGs together with 49 well-defined reference genotypes (same set of genotypes as in [18]), representing three parental species (*D. cucullata*, *D. galeata*, *D. longispina*) and their interspecific hybrids. It was confirmed that only *D. galeata* and *D. longispina* parental species were present in our dataset. Bayesian statistics were then used (in NewHybrids 1.1, [20]) to assign individuals to one of six possible classes (i.e., two parental species, F1 and F2 hybrids and both backcrosses). The probability threshold for assignments was set to 95% (10^6^ iterations after a burn-in of length 10^6^). All the following calculations of taxon-specific parameters are based on the taxon assignment in NewHybrids.

### Spatial patterns in taxon composition

To test if the taxonomic similarity of *Daphnia* communities corresponds with the geographical proximity of the lakes, the correlation between pairwise Euclidean distances based on the taxon composition and respective pairwise geographical distances between sampling sites was evaluated using a Mantel test (5000 permutations, Past v2.07, [41]). Two estimates of Euclidean distances were derived in Past v2.07 for the spring samples (i.e., April or May), based on either the frequency, or the presence/absence, of five *Daphnia* groups. These groups included all classes as identified by NewHybrids (i.e., *D. galeata, D. longispina*, F1 hybrids and F2 hybrids; backcrosses were not detected) and an additional group of individuals that could not be identified by this method. All ten lakes were included in these tests.

### Environmental preferences of parental taxa

A principal components analysis (PCA) of five environmental variables (i.e., lake age, lake size, maximum depth, total phosphorus concentration (P_tot_), nitrogen concentration (N)) and mean *Daphnia* density (see Additional file 2: Table S2) was used to relate *Daphnia* species occurrence to combinations of these parameters. Variables that did not conform to normality were log-transformed. To test if *Daphnia* species were nonrandomly distributed along each component of the PCA, a one-way ANOVA was used with the presence/absence of each *Daphnia* species (i.e., *D. galeata* or *D. longispina*) as a main factor. The species presence/absence category was assigned based on the whole-year dataset (Additional file 1: Table S1). All ten lakes were included in these tests.

### Temporal changes in taxon composition

The relative frequencies of five *Daphnia* groups (i.e., four classes: *D. galeata, D. longispina*, F1 hybrids and F2 hybrids identified by NewHybrids, and one group of individuals not identified by this method) were compared between spring (April or May) and autumn (October or November or, in the case of FELD, September), by applying a Monte Carlo approach with 10^5^ simulation runs [42]. Since separate tests were run for each of the seven lakes tested here (BOHM, FASA, FELD, HEIM, LERC, LUSS and WALD), sequential Bonferroni corrections [43] were applied when interpreting the results.

### Genetic distances among spatially and temporally isolated populations of parental species and hybrids

Pairwise Nei’s genetic distances [21] were calculated in GENALEX 6 [44], among populations belonging to *D. galeata*, *D. longispina* or hybrids (F1 and F2 NewHybrids classes were pooled into one group of “hybrids”, because of the low sample size of the F2 class). Population samples originating from different months were considered separately, and only populations with sample size N ≥ 6 were included. Then, genetic similarities among population samples were displayed using the Unweighted Pair-Group Method with Arithmetic Mean (UPGMA), as calculated in MEGA 4 [45].

### Gene flow and local production of hybrids

The number of migrants between lakes was calculated per generation (Nm) to estimate gene flow among populations of the two parental species (i.e., *D. galeata* and *D. longispina*) and the hybrids (F1 and F2 NewHybrids classes were pooled into one group of “hybrids”, because of the low sample size of the F2 class), respectively (in GENETIX 4.05). The whole-year dataset was pooled per taxon and lake. For each of the three taxa, only lake samples with N > 10 were considered to represent a local population and included in this analysis. Then, one-way ANOVA and Post-Hoc Tests were used to test for taxon-specific differences in the number of migrants (in SPSS 20.0).

In addition, it has been tested if hybrids are more likely formed by the parental species inhabiting their home lake (i.e., local production of hybrids) than by the parental species from other lakes. Thus, for each individual hybrid genotype, the log-likelihood with which it was assigned to parental groups (specific parental species from the home lake versus pooled populations of the same parental species from the other lakes) was calculated in Arlequin 3.0 [46]. To distinguish statistical differences in respective log-likelihoods of hybrid assignments, a paired t-test (10^3^ bootstrap replications) was run in SPSS 20.0. Although hybrids and respective parental species co-existed in FELD and LUSS, the test above could only be performed for LUSS hybrids, because only one hybrid genotype was detected in FELD.

### Test for clonality

To test the null hypothesis that an MLG encountered more than once was the result of sexual recombination, instead of clonal propagation, the *P*_sex_ value was calculated in GENCLONE 2.0 [47]. The calculations were performed individually for each abundant taxon and lake. Only individuals characterized at all 15 microsatellite loci (or with missing data at a single locus, SwiD2; many *D. longispina* individuals could not be amplified at this locus) were included in these and the follow-up analyses of clonal structure (i.e., 1856 of 1934 genotyped *Daphnia*, see Additional file 1: Table S1).

### Temporal changes in clonal composition

To test if differences in clonal composition increase with the time interval between samples, the clonal turnover rate was calculated between sampling dates (for all possible pairwise comparisons), per taxon and lake, by computing complements of the Morisita-Horn index (1-MH [48]; see also [49]) using the program SPADE [50] and 10^4^ bootstrap replications. This turnover rate takes into account not only changes in the presence of individual MLGs but also changes in MLG frequencies. Turnover rate varies from 0 (complete similarity) to 1 (no similarity). Then, the correlation between the pairwise turnover rate in clonal composition and the corresponding pairwise time differences between sampling dates (calculated in monthly intervals) was evaluated by a Mantel test (5000 permutations, Past v2.07). Finally, evidence was sought for the existence of an overall decrease in clonal diversity during the course of the growing season (i.e., clonal erosion). Clonal diversity was measured per taxon and sampling date as: (i) clonal richness R, and (ii) the inverse of the Simpson index. Clonal richness R was calculated as R = (G-1) / (N-1), where G is the number of genotypes and N represents sample size [51]. R varies from 0 (all individuals belong to one clone) to 1 (all individuals are different). The inverse of the Simpson index was calculated as 1/(∑P_i_^2^), where P_i_ is the proportion of the i^th^ clone. This index thus includes additional information about the distribution of clones within samples. The minimum value of this index is 1 (all individuals belong to one clone) whereas the maximum value is the number of unique clones in the sample. Linear regression analyses were applied to test for the effect of time on clonal diversity (i.e., either clonal richness R or the inverse of the Simpson index) using R-software [52]. The tests were run only on those populations for which samples spanning the entire growing season were available: *D. galeata* from FASA, *D. galeata* from LERC and *D. longispina* from WALD (a minimum sample size was set of 30 individuals).

## Competing interests

The authors declare that they have no competing interests.

## Authors’ contributions

SG and JW designed the study, MY carried out the molecular work and JG collected the environmental dataset. MY, SG and JW contributed to data analyses and preparation of the manuscript. All authors read and approved the final version.

## Supplementary Material

Additional file 1: Table S1 Sample size and clonal diversity of *Daphnia* populations inhabiting ten lakes in and around Munich. Individuals were genotyped at 15 microsatellite loci. Click here for file

Additional file 2: Table S2 Geographical location, physical and environmental characteristics of the ten lakes included in the present study. Click here for file

Additional file 3: Figure S1 Temporal changes in clonal composition within populations of: **(a)***D. galeata* from FASA, **(b)***D. galeata* from LERC and **(c)***D. longispina* from WALD (only in these three lakes were *Daphnia* communities present throughout the entire sampling season, i.e. from April to November 2011, and the sample size per analysed population ≥ 30). The frequencies of the most common clones (i.e. frequency ≥ 10% in at least one sample) are indicated by different shading, whereas rare clones were pooled into one category (white area, up to 100%). A blank square across the graph indicates a month when too few animals were available to calculate clonal frequencies. Clonal IDs were assigned based on variation at all 15 microsatellite loci (or 14 loci, if *Daphnia* could not be amplified at locus SwiD2). Click here for file
