# Supplementary material for: Hybridizing Daphnia communities from ten neighbouring lakes: spatio-temporal dynamics, local processes, gene flow and invasiveness
Source: BMC Evol Biol. 2014 Apr 12;14:80. doi: 10.1186/1471-2148-14-80 (PMC4101843; doi:10.1186/1471-2148-14-80)
Supplement: Additional file 1: Table S1 — Sample size and clonal diversity of Daphnia populations inhabiting ten lakes in and around Munich. Individuals were genotyped at 15 microsatellite loci. [file 1471-2148-14-80-S1.pdf]

Table S1. Sample size and clonal diversity of *Daphnia* populations inhabiting ten lakes in and around Munich. Individuals were genotyped at 15 microsatellite loci.

Lake (abbreviation)	Sampling month (of 2011)	Taxon[a]	N_1	N_2	MLG	Richness R[c]	Inverse of Simpson index[c]
Böhmerweiher (BOHM)	May	F1 hybrids	7	7	4	-	-
		non-identified[b]	1	1	1	-	-
	October	F1 hybrids	18	18	4	-	-
		non-identified	1	1	1	-	-
Fasanariesee (FASA)	April	*Daphnia galeata*	45	45	27	0.59	11.31
		non-identified	1	1	1	-	-
	May	*D. galeata*	47	47	17	0.35	3.29
		Daphnia longispina	1	1	1	-	-
	June	*D. galeata*	46	46	30	0.64	19.96
	July	*D. galeata*	42	41	27	0.65	18.89
	August	*D. galeata*	43	42	19	0.44	9.54
	September	*D. galeata*	85	78	35	0.44	16.90
	October	*D. galeata*	41	40	22	0.54	11.76
	November	*D. galeata*	42	39	30	0.76	22.70
		F2 hybrids	3	3	3	-	-
		non-identified	1	1	1	-	-
Feldmochinger See	May	F1 hybrids	46	46	1	0	1.00

(FELD)							
	September	*D. galeata*	79	72	40	0.55	17.51
Feringasee							
(FERI)	April	*D. galeata*	1	1	1	-	-
		F1 hybrids	43	43	9	0.19	1.77
		non-identified	1	1	1	-	-
	May	F1 hybrids	44	43	4	0.07	1.27
Heimstettner See							
(HEIM)	May	*D. galeata*	46	46	44	0.96	42.32
	September	*D. galeata*	20	17	17	-	-
	October	*D. galeata*	44	42	37	0.88	30.41
		F2 hybrids	2	0	na	-	-
	November	*D. galeata*	47	45	41	0.91	38.21
		F2 hybrids	1	1	1	-	-
Langwieder See							
(LANG)	April	*D. longispina*	47	42	29	0.68	19.60
	May	*D. longispina*	46	35	24	0.67	15.12
	June	*D. longispina*	44	40	23	0.56	10.81
Lerchenauer See							
(LERC)	April	*D. galeata*	45	45	25	0.55	12.58
		F1 hybrids	1	1	1	-	-
	May	*D. galeata*	47	47	30	0.63	17.67
	June	*D. galeata*	42	42	20	0.46	8.09
		F1 hybrids	3	3	2	-	-

		non-identified	1	1	1	-	-
	July	*D. galeata*	8	8	6	-	-
		F1 hybrids	1	0	na	-	-
	August	*D. galeata*	39	37	12	0.31	4.37
	September	*D. galeata*	33	31	17	0.53	11.38
		D. longispina	8	7	6	-	-
		non-identified	1	1	1	-	-
	October	*D. galeata*	39	39	25	0.63	12.57
	November	*D. galeata*	46	46	20	0.42	9.20
Lußsee							
(LUSS)	April	*D. longispina*	14	13	13	-	-
		F1 hybrids	10	10	3	-	-
		F2 hybrids	2	1	1	-	-
	May	*D. longispina*	42	42	25	0.59	13.36
		F1 hybrids	2	2	2	-	-
	June	*D. longispina*	45	45	34	0.75	27.74
		F1 hybrids	2	1	1	-	-
	July	*D. longispina*	20	20	12	-	-
		F1 hybrids	1	1	1	-	-
	August	*D. longispina*	42	41	27	0.65	13.67
		F1 hybrids	1	1	1	-	-
	September	*D. longispina*	23	23	15	-	-
		F1 hybrids	2	2	1	-	-
		F2 hybrids	1	1	1	-	-

	November	*D. longispina*	31	30	25	0.82	21.43
		F1 hybrids	1	1	1	-	-
		F2 hybrids	5	4	4	-	-
		non-identified	1	1	1	-	-
Olchinger See (OLCH)	April	*D. longispina*	47	46	39	0.84	33.06
	May	*D. longispina*	39	36	33	0.91	30.85
Waldschwaigsee (WALD)	April	*D. longispina*	45	43	20	0.45	8.29
	May	*D. longispina*	46	43	22	0.50	8.29
	June	*D. longispina*	46	46	28	0.60	17.34
	July	*D. longispina*	45	44	26	0.58	14.24
		non-identified	1	1	1	-	-
	August	*D. longispina*	43	43	26	0.60	15.28
	September	*D. longispina*	44	44	27	0.60	22.00
	October	*D. longispina*	40	38	24	0.62	16.41
		F2 hybrids	1	0	na	-	-
	November	*D. longispina*	40	39	22	0.55	8.22
		F2 hybrids	1	0	na	-	-
		non-identified	2	1	1	-	-
Total			1934	1856			

N_1: maximum sample size, including individuals with some missing loci; N_2: sample size including only those individuals which were successfully characterized at all 15 microsatellite loci (or at 14 loci, in case of a missing SWiD2 locus; see the main text); MLG: number of multilocus genotypes (calculated from N_2); [a] taxon membership was defined by the NewHybrids software; [b] could not be assigned when applying a probability threshold of 95% in NewHybrids; [c] only calculated if $N_2 \geq 30$; na, not applicable.
