# Supplementary material for: Hybridizing Daphnia communities from ten neighbouring lakes: spatio-temporal dynamics, local processes, gene flow and invasiveness
Source: BMC Evol Biol. 2014 Apr 12;14:80. doi: 10.1186/1471-2148-14-80 (PMC4101843; doi:10.1186/1471-2148-14-80)
Supplement: Additional file 2: Table S2 — Geographical location, physical and environmental characteristics of the ten lakes included in the present study. [file 1471-2148-14-80-S2.pdf]

Table S2. Geographical location, physical and environmental characteristics of the ten lakes included in the present study.

Lake	Longitude	Latitude	Age (years)	Surface area (km^2)	Max depth (m)	N^a (mg/l)	P_{tot}^a (mg/l)	mean *Daphnia* density (individuals/L)
Böhmerweiher	11°22.784'E	48°10.449'N	46	0.01	3	6.15	0.010	0.02
Fasanariesee	11°31.770'E	48°12.250'N	76	0.14	11	2.71	0.022	1.64
Feldmochinger See	11°30.881'E	48°12.831'N	76	0.16	5	3.22	0.030	0.03
Feringasee	11°40.297'E	48°11.684'N	36	0.32	7	4.02	0.004	0.03
Heimstettner See	11°44.314'E	48°09.351'N	73	0.11	5	4.98	0.009	0.60
Langwieder See	11°24.881'E	48°11.700'N	73	0.18	8	4.70	0.005	0.55
Lerchenauer See	11°32.232'E	48°11.838'N	76	0.08	7	2.30	0.026	2.16
Lußsee	11°25.123'E	48°11.910'N	11	0.17	16	3.64	0.005	0.09
Olchinger See	11°21.432'E	48°12.539'N	71	0.14	6	6.46	0.005	0.02
Waldschwaigsee	11°26.272'E	48°13.507'N	39	0.085	15	5.79	0.004	0.87

[a] measurements were taken on the following dates: BOHM: 26.04.2004; FASA: 08.03.2001; FELD: 31.03.2009; FERI: 11.04.2005; HEIM :24.04.2013; LANG:22.04.2010; LERC: 31.03.2009; LUSS: 22.04.2010; OLCH: 24.03.2011;WALD: 17.04.2013. N: nitrogen concentration. P_{tot}: total phosphorus concentration.
