# Supplementary figures and images for: Hybridizing Daphnia communities from ten neighbouring lakes: spatio-temporal dynamics, local processes, gene flow and invasiveness
Source: BMC Evol Biol. 2014 Apr 12;14:80. doi: 10.1186/1471-2148-14-80 (PMC4101843; doi:10.1186/1471-2148-14-80)

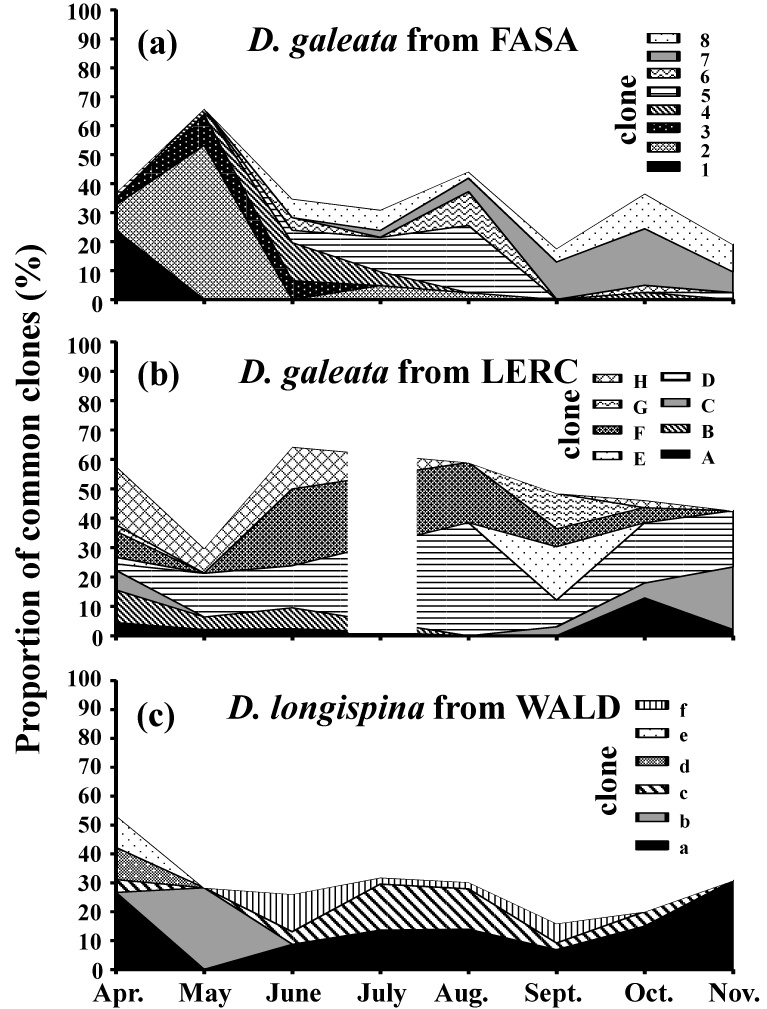

Supplement: Additional file 3: Figure S1 — Temporal changes in clonal composition within populations of: (a)D. galeata from FASA, (b)D. galeata from LERC and (c)D. longispina from WALD (only in these three lakes were Daphnia communities present throughout the entire sampling season, i.e. from April to November 2011, and the sample size per analysed population ≥ 30). The frequencies of the most common clones (i.e. frequency ≥ 10% in at least one sample) are indicated by different shading, whereas rare clones were pooled into one category (white area, up to 100%). A blank square across the graph indicates a month when too few animals were available to calculate clonal frequencies. Clonal IDs were assigned based on variation at all 15 microsatellite loci (or 14 loci, if Daphnia could not be amplified at locus SwiD2). [file 1471-2148-14-80-S3.tiff]
